# Image-Based Multi-Target Tracking through Multi-Bernoulli Filtering with Interactive Likelihoods

**DOI:** 10.3390/s17030501

**Published:** 2017-03-03

**Authors:** Anthony Hoak, Henry Medeiros, Richard J. Povinelli

**Affiliations:** Department of Electrical & Computer Engineering, Marquette University, 1551 W. Wisconsin Ave., Milwaukee, WI 53233, USA; anthony.hoak@marquette.edu (A.H.); richard.povinelli@marquette.edu (R.J.P.)

**Keywords:** multi-target tracking, multi-Bernoulli filter, sequential Monte Carlo

## Abstract

We develop an interactive likelihood (ILH) for sequential Monte Carlo (SMC) methods for image-based multiple target tracking applications. The purpose of the ILH is to improve tracking accuracy by reducing the need for data association. In addition, we integrate a recently developed deep neural network for pedestrian detection along with the ILH with a multi-Bernoulli filter. We evaluate the performance of the multi-Bernoulli filter with the ILH and the pedestrian detector in a number of publicly available datasets (2003 PETS INMOVE, Australian Rules Football League (AFL) and TUD-Stadtmitte) using standard, well-known multi-target tracking metrics (optimal sub-pattern assignment (OSPA) and classification of events, activities and relationships for multi-object trackers (CLEAR MOT)). In all datasets, the ILH term increases the tracking accuracy of the multi-Bernoulli filter.

## 1. Introduction

Multi-target tracking (MTT) is a well-researched problem, with a history going back over 50 years [1]; however, it remains an open research problem [2,3]. It has many applications, including aviation [4] and air traffic control [5], ballistic missile defense [6,7], smart surveillance [8,9], robotics [10] and autonomous vehicles [11,12,13,14,15]. The goal of MTT is to simultaneously estimate both the number of targets and their states (position, size, velocity, etc.) through time [16]. This can be a difficult task for a number of different reasons; to name just a select few of these challenges: (1) mathematically-consistent ways of defining and using estimation error; (2) mathematically modeling targets entering and leaving the scene (target births and deaths); (3) the task of data association (associating targets with correct measurements, as well as associating targets temporally); and (4) robustness to different and dynamic scenarios.

In image-based tracking, measurements are in the form of individual image frames, and the objective is to track the targets and their states through the image sequence. It is often the case that multiple measurement observations are made within a given image frame, and it is necessary to associate these measurements with targets or tracks. Tracks are defined by Ristic et al. [16] as a “labeled temporal sequence of state estimates associated with the same target” (the terms “tracks” and ”trajectories” are used interchangeably in this article).

In this paper, an interactive likelihood (ILH) for the multi-Bernoulli filter (MBF) (the abbreviation MBF is used for the multi-Bernoulli filter as the implementation is slightly different from the common MeMBer [17] and CB-MeMBer [18] variations) is developed and evaluated. The purpose of the ILH is to reduce the need for data association, addressing Challenge 3. It is based entirely within the Bayesian random finite set (RFS) framework and therefore does not require any external data association mechanism(s). The proposed approach is a novel method for addressing the fundamental data association issue in the field of multi-target tracking [4]. The Bayesian-RFS framework simultaneously handles Challenges 1 and 2 elegantly, and much progress has been made in recent years to also address Challenge 3 using this framework in a mathematically-rigorous way through the labeled multi-Bernoulli filter and its variants [19,20,21,22,23,24]. Our work corresponds to another important step in that direction.

In order to address Challenge 4, a state-of-the-art deep neural network for pedestrian detection is integrated into the MBF with the ILH. To the best of our knowledge, there has been no work on integrating deep networks in a track-before-detect Bayesian-RFS framework. Because deep networks achieve such promising results in object and pedestrian detection and of the lack of existing research in using these detectors with RFS approaches, there is substantial unexplored potential in the combination of these two state-of-the-art techniques.

Specifically, the main contributions presented in this paper are as follows:

1. a novel interactive likelihood (ILH) method for sequential Monte Carlo (SMC) image-based trackers that can be computed non-iteratively to preclude the tracker from sampling from areas that belong to different targets;
2. this interactive likelihood method is integrated with the multi-Bernoulli filter, a state-of-the-art RFS tracker, which is referred to as MBFILH;
3. the deep learning technique for pedestrian detection proposed in [25] is combined with the MBFILH; and
4. an extensive evaluation is carried out using several publicly available datasets and standard evaluation metrics.

The rest of this paper is organized as follows: Section 2 discusses related works, including a brief description of common multi-target tracking algorithms, as well as highlighting recent trends. In Section 3, we present necessary background information, notation and definitions. We develop our ILH in Section 4. In Section 5 we evaluate the ILH in a number of different datasets, report numerical results and provide a brief discussion of these results. Finally, in Section 6, we briefly summarize our contribution.

## 2. Related Work

In this section, we present a general discussion of multi-target tracking including standard techniques and challenges and also identify a trend in the current research within the multi-target tracking community.

### 2.1. Common Multi-Target Tracking Algorithms

Many different MTT methods have been proposed over the years, but the most mature and most common algorithms are the joint probabilistic data association filter (JPDAF) [26] and the multiple hypothesis tracking (MHT) method [4]. In the last decade [1], there has been substantial research in the use of random finite sets within a Bayesian framework for multi-target tracking [27,28,29,30,31,32,33,34]. One benefit to RFS-based approaches is that they have mathematically consistent and rigorous ways for handling estimation error and target births and deaths. In other words, they are well equipped to handle Challenges 1 and 2 listed in the first paragraph of Section 1. In the last two years, there has been a resurgence of detection-based approaches, including new variations of the more mature multi-target tracking algorithms, for example a revisiting of the JPDAF [35]. Most recently, due to the vast amount of research on (deep) neural networks for object and pedestrian detection [25,36,37,38,39,40,41,42,43], track-by-detection multi-target tracking approaches are starting to be proposed that make use of these state-of-the-art detectors [44].

The process of associating measurements with appropriate targets or tracks is known as data association. Historically, it is one of the major challenges in multi-target tracking [4] and is still an active research topic [45,46,47]. It is desirable for targets to remain separated within the image and for no occlusions to take place; however, this is often not the case. For example, in sport player tracking situations, targets often are in close proximity, and occlusions are frequent. This type of situation can lead to numerous, ambiguous measurements, where data association becomes especially important. If the data association process is not handled adequately, target tracks or labels may be switched or dropped entirely, resulting in overall tracking inaccuracy.

The two classical multi-target tracking algorithms, JPDAF and MHT, have internal mechanisms to accomplish the data association task. However, traditional RFS approaches do not have such mechanisms in place and, therefore, often require an additional layer of complexity external to the RFS framework in order to perform data association. For example, in [27], a graph theoretical approach (based on the work of Shafique and Shah in [47]) is used in conjunction with a multi-Bernoulli filter. The significant recent contributions on the labeled multi-Bernoulli filter correspond to additional attempts to overcome this limitation [19,20,21,22,23,24].

There are disadvantages to all of the most common multi-target tracking algorithms. Both the JPDAF and the MHT require an exponential number of terms as time progresses in order to solve the data association problem [48]. Theoretically, RFS methods should not require an additional, external technique for data association; however, in practice, these trackers need to be implemented using Monte Carlo approaches, and the sampling process introduces confusion that may cause incorrect associations. Hence, in practice, data association is strictly necessary for multi-target tracking, even when RFS approaches are employed, and is also still one of the most limiting factors.

To address this fundamental issue, an interactive likelihood for the multi-Bernoulli filter is presented. The interactive likelihood technique exploits the spatial information that exists in any given image observation and reduces the need for data association. It works by modifying the sequential Monte Carlo sampling process so that the spatial probability distributions of nearby similar targets do not overlap. As a result, it reduces the confusion among these targets and, hence, avoids estimation errors in these challenging scenarios.

### 2.2. Current Trends

Most multi-target tracking algorithms can also be classified as either track-by-detection or track-before-detection. Track-by-detection algorithms use post-processed data, that is the raw sensor measurements have had some kind of thresholding performed [49]. There is a vast amount of work currently being done on developing and evaluating new algorithms (since 2015) that fall into the former category [3,50,51,52,53,54]. In track-by-detection approaches, there are often separate techniques for detection and data association. In these approaches, detections are typically obtained by a scanning algorithm that searches the entire image and determines where targets are likely to be located. These detections are then processed by a separate algorithm for association. This effectively splits the multi-target tracking problem into two separate problems: detection and association. In fact, in the most recently-proposed multi-target tracking benchmarks [55,56], it is encouraged to use ‘standard’ detections to remove the problem of detection entirely and focus completely on association. This means that new multi-target tracking methods can be proposed, which are incapable of producing measurements from an image and therefore do not really even need to process images at all, but must only be able to use these standard detections. This allows for relatively quick evaluations in large datasets with large image sizes. While this paradigm is beneficial for the sake of fairly comparing all track-by-detection approaches, it has a number of other effects: (1) it removes all image processing from the task, and thus, it is no longer a computer vision problem; (2) it becomes difficult for sequential Monte Carlo approaches to be included in the benchmarks, as Monte Carlo evaluations are necessary and numerous trials must be performed, taking significantly longer in large datasets than using standard, precomputed detections; (3) all track-before-detection approaches are also at a disadvantage, as they solve both the detection and tracking problems simultaneously, adding more opportunities for error; and (4) it assumes that detectors have plateaued in performance, which may inadvertently reduce the amount of research to develop new and higher performing detectors.

In situations in which target detection itself is challenging, such as in scenarios with substantial clutter, track-before-detect methods tend to perform significantly better [57,58,59]. The contribution presented in this paper, hence, falls into the second category, track-before-detection, in which there are no scanning or searching schemes involved, and detections are not strictly necessary. This method is also considered “online”, as new estimates about the current multi-target state are available at each time instant.

## 3. Method

In this section, we provide some necessary background for presenting our method. We briefly discuss the image-based multi-Bernoulli filter, its corresponding Bayes’ recursion and describe a particle filter implementation.

### 3.1. Image-Based Multi-Bernoulli Filter

The interactive likelihood presented in this paper is constructed within the multi-Bernoulli filter presented by Hoseinnezhad et al. in [27]. Therefore, the notation used for the multi-target Bayes’ and multi-Bernoulli filter will be similar. Prior to describing the interactive likelihood and its development, it is necessary to establish a number of definitions. A single image of *m* total pixels is represented by a one-dimensional vector

(1) $$y={\left[\begin{array}{ccc} y_{0} & \cdots & y_{m} \end{array}\right]}^{T}.$$

For a single image within an image sequence, let the number of targets be *n* and their states be $x_{1},x_{2},\cdots x_{n}$. In this paper, the state of each target $x_{i}$ consists of its horizontal *u* and vertical *v* coordinates in the image, as well as its height *h* and width *w*, such that:

(2) $$x_{i}={\left[\begin{array}{cccc} u & v & h & w \end{array}\right]}^{T}.$$

Then, the multi-target state is represented as a finite set:

(3) $$X=\{x_{1},x_{2},\cdots x_{n}\}.$$

The defining feature of the multi-Bernoulli filter is the multi-Bernoulli RFS, which is a union of *M* independent Bernoulli RFSs. The probability density of a Bernoulli RFS $X^{\left(i\right)}$ is (see [17,31] for details regarding Bernoulli and multi-Bernoulli RFSs):

(4) $$\pi \left(X^{\left(i\right)}\right)=\left\{\begin{array}{cc} 1-r, & \mathrm{if}\;X^{\left(i\right)}=\emptyset \\ r\cdot p(\cdot ), & \mathrm{if}\;X^{\left(i\right)}=\left\{x_{i}\right\}, \end{array}\right.$$

where *r* is the probability of existence for $x_{i}$, the only element of *X* if it is non-empty, and $x_{i}$ is distributed according to the probability density p(·). $X^{\left(i\right)}$ has the probability 1−r of being empty. Then, a multi-Bernoulli RFS *X* is the union of *M* independent Bernoulli RFSs $X^{\left(i\right)}$:

(5) $$X=\overset{M}{\underset{i=1}{⋃}}X^{\left(i\right)}$$

and is fully characterized by the parameter set ${\left\{\left(r^{\left(i\right)},p^{\left(i\right)}\left(\cdot \right)\right)\right\}}_{i=1}^{M}$ [27].

### 3.2. Bayes’ Recursion

Detailed discussions of the Bayes’ recursion and the multi-Bernoulli filter can be found in [27,28,29,30,32]. Only the essential information will be given here. For a given multi-target state *X* and image sequence $y_{1:k-1}$, the multi-target Bayes’ filter computes the predicted state $\pi_{k|k-1}\left(X_{k}|y_{1:k-1}\right)$ by propagating the multi-target posterior $\pi_{k-1}\left(\cdot |y_{1:k-1}\right)$ from time step k−1 to *k* through the multi-target transition density $f_{k|k-1}\left(\cdot |\cdot \right)$. Once a measurement is available, the posterior at time *k*, $\pi_{k}\left(X_{k}|y_{1:k}\right)$, is computed using the multi-target likelihood function g(·|·) (see Mahler’s FISST [60,61] for more detailed information on the set integrals required to perform these steps). The Bayes’ recursion is, in general, intractable [17,29,30,31,32], and therefore, approximations are necessary. We adopt the commonly-used multi-target transition density discussed in [27,29], such that at time k−1, the elements $x_{k-1}$ of the multi-target state $X_{k-1}$ either continue to exist at time *k* with probability $p_{S,k}\left(x_{k-1}\right)$ and transition to state $x_{k}$ with probability density $f_{k|k-1}\left(x_{k}|x_{k-1}\right)$ or die with probability $1-p_{S,k}\left(x_{k-1}\right)$. The behavior of a target with state $x_{k-1}$ at time k−1 can then be modeled by $S_{k|k-1}\left(x_{k-1}\right)$, which are the Bernoulli RFSs corresponding to each target that survived at time instant *k* [29]. At time *k*, the multi-target state $X_{k}$ is then:

(6) $$X_{k}=\underset{x_{k-1}\in X_{k-1}}{⋃}S_{k|k-1}\left(x_{k-1}\right)\cup \Gamma_{k}.$$

The RFS $\Gamma_{k}={\left\{\left(r_{\Gamma ,k}^{\left(i\right)},p_{\Gamma ,k}^{\left(i\right)}\left(\cdot \right)\right)\right\}}_{i=1}^{M_{\Gamma ,k}}$ contains all of the targets born at time *k*. The likelihood of an image observation *y* given the multi-target state *X* is:

(7) $$g\left(y|X\right)=\prod_{i=1}^{n}g_{f}\left(y_{x_{i}}\right),$$

where $g_{f}\left(y_{x_{i}}\right)$ is the likelihood that a target with state $x_{i}$ is present in image *y*.

### 3.3. Likelihood Functions

We first employ the same likelihood function $g_{f}\left(y_{x_{i}}\right)$ as [27], and more detailed information can be found there regarding how it is constructed. For the scope of this paper, it is sufficient to say that it is based on hue-saturation-value (HSV) histograms, and the training data consist of $n_{train}=850$ references histograms to which target histograms are compared. It has the form:

(8) $$g_{f}\left(v_{i}\right)=\frac{\zeta }{n_{train}h^{N}}\sum_{j=1}^{n_{train}}\kappa \left(\frac{d(v_{i},v_{j}^{*})}{h}\right),$$

where *ζ* is a normalization constant, $v_{i}$ is the target histogram vector of *y*, ${\left\{v_{j}^{*}\right\}}_{j=1}^{n_{train}}$ is the set of reference histograms, κ(·) is a Gaussian kernel function, *h* is the kernel bandwidth and *N* is the number of histogram bins. The difference between histograms is measured using the Bhattacharyya distance, given by:

(9) $$d\left(v_{i},v_{j}^{*}\right)={\left(1-\sum_{r=1}^{N}\sqrt{v_{j}^{*}\left(r\right)v_{i}\left(r\right)}\right)}^{1/2}.$$

We consider a second, simple, but more general likelihood function based on the pedestrian detector presented in [25]:

(10) $$g_{f}\left(y_{x_{i}}\right)=\gamma PD{\left(y_{x_{i}}\right)}^{2},$$

where $PD(y_{x_{i}})$ is the output of the pedestrian detector for the image observation $y_{x_{i}}$ and *γ* is a scalar coefficient. More information about the pedestrian detector will be presented in Section 4.1.

### 3.4. Particle Filter Implementation

RFS filters (including the multi-Bernoulli) are often implemented using sequential Monte Carlo (SMC) methods, such as the particle filter [27,28,30,32,33,62]. We use the particle filter implementation used by Hoseinnezhad et al. in [27], which is similar to those used in [29,30]. In order to translate the multi-Bernoulli recursion to a particle filter implementation, let the proposal densities $q_{k}^{\left(i\right)}\left(\cdot |x_{k-1},y_{k}\right)$ and $b_{k}^{\left(i\right)}\left(\cdot |y_{k}\right)$ be known and the probability density $p_{k-1}^{\left(i\right)}\left(\cdot \right)$ be given by a set of weighted samples (particles):

(11) $$p_{k-1}^{\left(i\right)}\left(x\right)=\sum_{j=1}^{L_{k-1}^{\left(i\right)}}w_{k-1}^{\left(i,j\right)}\delta_{x_{k-1}^{\left(i,j\right)}}\left(x\right).$$

Furthermore, suppose that at time k−1, the multi-target multi-Bernoulli posterior $\pi_{k-1}$ is known, then the multi-Bernoulli parameters for the predicted target state can be calculated as follows:

(12) $$r_{k|k-1}^{\left(i\right)}=r_{k-1}^{\left(i\right)}\sum_{j=1}^{L_{k-1}^{\left(i\right)}}w_{k-1}^{\left(i,j\right)}p_{S,k}\left(x_{k-1}^{\left(i,j\right)}\right).$$

(13) $$p_{k|k-1}^{\left(i\right)}\left(x\right)=\sum_{j=1}^{L_{k-1}^{\left(i\right)}}{\tilde{w}}_{k|k-1}^{\left(i,j\right)}\delta_{x_{k|k-1}^{\left(i,j\right)}}\left(x\right).$$

(14) $$r_{\Gamma ,k}^{\left(i\right)}=\text{birth model parameter}.$$

(15) $$p_{\Gamma ,k}^{\left(i\right)}\left(x\right)=\sum_{j=1}^{L_{\Gamma ,k}^{\left(i\right)}}{\tilde{w}}_{\Gamma ,k}^{\left(i,j\right)}\delta_{x_{\Gamma ,k}^{\left(i,j\right)}}\left(x\right),$$

where:

(16) $$x_{k|k-1}^{\left(i,j\right)}∼q_{k}^{\left(i\right)}\left(\cdot |x_{k-1}^{\left(i,j\right)},y_{k}\right),\;\mathrm{for}\;j=1,\cdots ,L_{k|k-1}^{\left(i\right)},$$

(17) $$w_{k|k-1}^{\left(i,j\right)}=\frac{w_{k-1}^{\left(i,j\right)}f_{k|k-1}\left(x_{k|k-1}^{\left(i,j\right)}|x_{k-1}^{\left(i,j\right)}\right)p_{S,k}\left(x_{k-1}^{\left(i,j\right)}\right)}{q_{k}^{\left(i\right)}\left(x_{k|k-1}^{\left(i,j\right)}|x_{k-1}^{\left(i,j\right)}\right)},$$

(18) $${\tilde{w}}_{k|k-1}^{\left(i,j\right)}=\frac{w_{k|k-1}^{\left(i,j\right)}}{\sum_{j=1}^{L_{k|k-1}^{\left(i\right)}}w_{k|k-1}^{\left(i,j\right)}},$$

(19) $$x_{\Gamma ,k}^{\left(i,j\right)}∼b_{k}^{\left(i\right)}\left(\cdot |y_{k}\right)\;\mathrm{for}\;j=1,\cdots ,L_{\Gamma ,k}^{\left(i\right)},$$

(20) $$w_{\Gamma ,k}^{\left(i,j\right)}=\frac{p_{\Gamma ,k}\left(x_{\Gamma ,k}^{\left(i,j\right)}\right)}{b_{k}^{\left(i\right)}\left(x_{\Gamma ,k}^{\left(i,j\right)}|y_{k}\right)},$$

(21) $${\tilde{w}}_{\Gamma ,k}^{\left(i,j\right)}=\frac{w_{\Gamma ,k}^{\left(i,j\right)}}{\sum_{j=1}^{L_{\Gamma ,k}^{\left(i\right)}}w_{\Gamma ,k}^{\left(i,j\right)}}.$$

Once the multi-Bernoulli parameters have been predicted, that is $\pi_{k|k-1}={\left\{\left(r_{k|k-1}^{\left(i\right)},p_{k|k-1}^{\left(i\right)}\left(\cdot \right)\right)\right\}}_{i=1}^{M_{k|k-1}}$ is known, the updated multi-Bernoulli parameters can be computed as follows:

(22) $$r_{k}^{\left(i\right)}=\frac{r_{k|k-1}^{\left(i\right)}\varrho_{k}^{\left(i\right)}}{1-r_{k|k-1}^{\left(i\right)}+r_{k|k-1}^{\left(i\right)}\varrho_{k}^{\left(i\right)}},$$

(23) $$p_{k}^{\left(i\right)}=\frac{1}{\varrho_{k}^{\left(i\right)}}\sum_{j=1}^{L_{k|k-1}^{\left(i\right)}}w_{k|k-1}^{\left(i,j\right)}g_{y_{k}}\left(x_{k|k-1}^{\left(i,j\right)}\right)\delta_{x_{k|k-1}^{\left(i,j\right)}}\left(x\right),$$

(24) $$\varrho_{k}^{\left(i\right)}=\sum_{j=1}^{L_{k|k-1}^{\left(i\right)}}w_{k|k-1}^{\left(i,j\right)}g_{y_{k}}\left(x_{k|k-1}^{\left(i,j\right)}\right).$$

## 4. Interactive Likelihood

**Figure 1:**
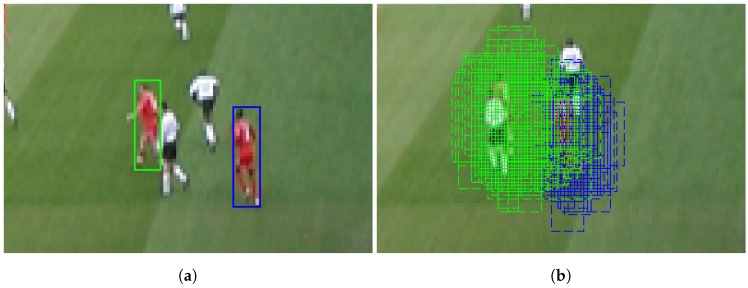
These two images illustrate the sensitivity of sequential Monte Carlo (SMC) methods to clutter. (**a**) Two targets tracked by a particle filter implementation of a multi-Bernoulli filter. The solid green and blue rectangles represent the estimated positions of the targets; (**b**) The same targets being tracked while particles are visible. Dashed rectangles represent the particles. Note how some particles of the target on the left (green) overlap and sample the target on the right (blue) (original images obtained from the 2003 PETS INMOVE dataset).

A fundamental requirement of the multi-Bernoulli filter is that targets remain completely separated within the image [27,29,32,33], that is they should not occlude one another. However, this is rarely the case, and in most applications, targets are often in close proximity and occlusions frequent. SMC implementations are especially sensitive to this requirement as particles are not inherently associated with a given target. Therefore, when multiple targets are in close enough proximity, the particles of one target are influenced by the other (see Figure 1 for an example of this happening). What defines “close enough proximity” can depend on any number of parameters, and it is often application specific.

One way to mitigate this effect is to weigh down the likelihood of the particles as they approach particles corresponding to other existing targets and then incorporate this weighting into the standard likelihood calculation of the multi-Bernoulli filter (Equation (7)). This approach is inspired by the work done by Qu et al. in [63], where distributed particle filters were used to separately track multiple targets, and an interactive likelihood was developed based on observation distances. It is also conceptually similar to the occlusion handling heuristics proposed by Xiao and Oussalah in [64] and by Yang and Yang in [65]. However, there are two major differences between these works and our approach: (1) our interactive likelihood is based on particle distances, instead of the estimated target positions; and (2) instead of keeping track of each target separately, we use a common RFS-based multi-target tracker, the multi-Bernoulli filter of [27]. An undesirable consequence of the use of the estimated target positions in [63] is that changing the particles causes the estimated position to change, which in turn changes the particle positions. This iterative estimation can be avoided by using particle distances directly as proposed here.

**Figure 2:**
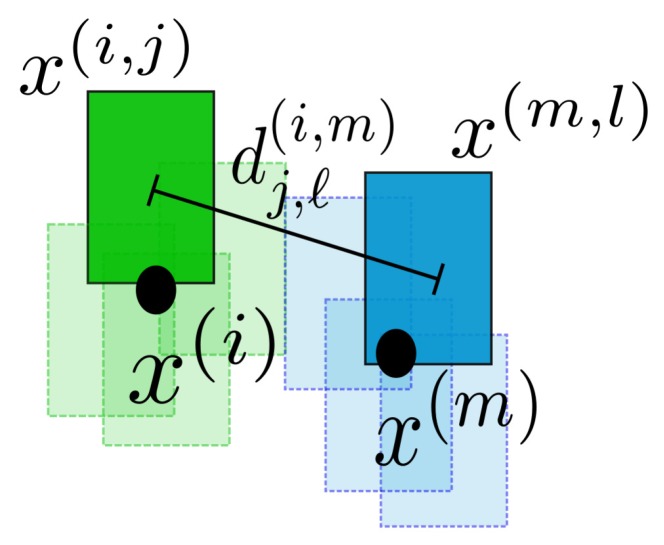
The distance between particle $x^{\left(i,j\right)}$ of target $x^{\left(i\right)}$ and particle $x^{\left(m,\ell \right)}$ of target $x^{\left(m\right)}$. The green and blue rectangles represent the particles associated with the two targets. The centers of the estimated target positions are represented by the black circles.

The first step in constructing the interactive likelihood is to associate particles with targets. This requires augmenting the state of the target with a unique label. Particles are then associated with a label, and distances can then be computed for particles associated with a given label to the particles associated with all other labels. The distance between the *j*-th particle of target $x^{\left(i\right)}$, denoted $x^{\left(i,j\right)}$, and the *ℓ*-th particle of target $x^{\left(m\right)}$, denoted $x^{\left(m,\ell \right)}$, is calculated in the image plane using the Euclidean distance:

(25) $$d_{j,\ell }^{\left(i,m\right)}\left(x^{\left(i,j\right)},x^{\left(m,\ell \right)}\right)=\sqrt{{\left(u^{\left(i,j\right)}-u^{\left(m,\ell \right)}\right)}^{2}+{\left(v^{\left(i,j\right)}-v^{\left(m,\ell \right)}\right)}^{2}},$$

where $u^{\left(\cdot \right)}$ and $v^{\left(\cdot \right)}$ are respectively the horizontal and vertical pixel coordinates of the centroid of the corresponding particle rectangle (see Figure 2 for an illustration of this distance). We intentionally refrain from using the 4D distance (that is, we do not include the width and height of the target) because we want to avoid confusion between target samples regardless of the relative target sizes. It should be noted that the case where i=m corresponds to the distance from a given target to itself and therefore does not need to be calculated. This distance is then used within the interactive likelihood weighting function.

Suppose that between the prediction step at time k−1 and the update step to time *k*, the cardinality of the multi-target state *X* is *M*. Let the number of particles associated with a given target $x^{\left(i\right)}$ be $L^{\left(i\right)}$. The interactive likelihood weight $\alpha^{\left(i,j\right)}$ for the *j*-th particle of target $x^{\left(i\right)}$ is determined by the interactive likelihood weighting function (note that the time subscript is dropped for notational convenience, but these calculations must be performed during each time step):

(26) $$\alpha^{\left(i,j\right)}\left(x^{\left(i,j\right)}\right)=\underset{m\neq i}{\prod_{m=1}^{M}}\prod_{\ell =1}^{L^{\left(m\right)}}1-\zeta e^{\frac{-{\left(d_{j,\ell }^{\left(i,m\right)}\left(x^{\left(i,j\right)},x^{\left(m,\ell \right)}\right)\right)}^{2}}{\sigma^{2}}}.$$

**Figure 3:**
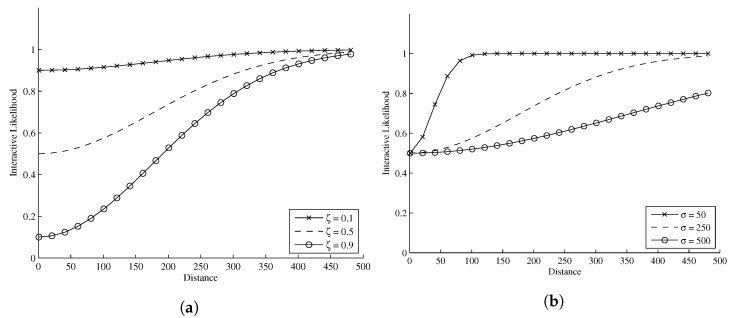
(**a**) Effect of changing *ζ* while keeping *σ* = 250. The *x*-axis represents distance (in pixels), and the *y*-axis is the corresponding interactive likelihood function value. The top, middle and lower lines correspond to *ζ* values of 0.1, 0.5 and 0.9, respectively; (**b**) Effect of changing *σ* while keeping *ζ* = 0.5. The *x*-axis represents distance (in pixels), and the *y*-axis is the corresponding interactive likelihood function value. The top, middle and lower lines correspond to *σ* values of 50, 250, and 500, respectively.

Both *ζ* and *σ* can be considered tuning parameters. The threshold at which the interactive likelihood starts to influence particle weights is determined by *σ*; it defines what is ‘close enough proximity’ and corresponds to the (pixel) distance at which targets start to influence one another. The higher the value of *σ*, the greater the distance of the influence of the interactive likelihood. The intensity of the influence is determined by *ζ*. These two parameters can be adjusted to obtain the desired particle interaction behavior for a given application. Figure 3a,b illustrates, in a 2D example, how changing both *σ* and *ζ* affect the interactive likelihood magnitude. The interactive likelihood term $\alpha^{\left(i,j\right)}$ can then be integrated into the particle filter update steps Equations (23) and (24) by simply multiplying the standard likelihood $g_{y_{k}}\left(x_{k|k-1}^{\left(i,j\right)}\right)$ term by the interactive likelihood term $\alpha^{\left(i,j\right)}\left(x_{k|k-1}^{\left(i,j\right)}\right)$,

(27) $$p_{k}^{\left(i\right)}=\frac{1}{\varrho_{k}^{\left(i\right)}}\;\sum_{j=1}^{L_{k|k-1}^{\left(i\right)}}w_{k|k-1}^{\left(i,j\right)}g_{y_{k}}\left(x_{k|k-1}^{\left(i,j\right)}\right)\cdot \alpha^{\left(i,j\right)}\left(x_{k|k-1}^{\left(i,j\right)}\right)\delta_{x_{k|k-1}^{\left(i,j\right)}}\left(x\right),$$

(28) $$\varrho_{k}^{\left(i\right)}=\sum_{j=1}^{L_{k|k-1}^{\left(i\right)}}w_{k|k-1}^{\left(i,j\right)}g_{y_{k}}\left(x_{k|k-1}^{\left(i,j\right)}\right)\alpha^{\left(i,j\right)}\left(x_{k|k-1}^{\left(i,j\right)}\right).$$

It should be noted that Equation (22) also changes appropriately.

### 4.1. Deep Learning for Pedestrian Detection

We integrate the deep learning technique for pedestrian detection described in [25] with the multi-Bernoulli filter, with and without the ILH. The deep network consists of five layers (in order): (1) a convolutional layer; (2) a layer for average pooling; (3) a second convolutional layer; (4) a deformation layer; and finally, (5) a visibility reasoning and classification layer. The 2009 Caltech pedestrian detection dataset [66] was used for the training of the network.

As mentioned in Section 3.2, we use the pedestrian detector as a likelihood function, and therefore, the algorithm still retains the track-before-detect characteristic. This is accomplished by feeding the network with each particle associated with a given target and using the output of the network straightforwardly as the likelihood of that particle. Therefore, Equations (27) and (28) remain the same; however, the likelihood function is now given by Equation (10) instead of Equation (8). We refer to the MBF and MBFILH with the pedestrian detector (PD) likelihood function as MBF PD and MBFILH PD, respectively.

In order to use the pedestrian detector, it is necessary to preprocess the input data (the randomly-sized particles/samples from the multi-Bernoulli filter shown as dashed-line rectangles in Figure 1). Each particle is first converted to YUVcolor space and resized to 84 × 28; hence, all three input channels require an image of an overall size 84 × 28. For Channel 2, the overall dimensions of 84 × 28 are achieved by concatenating three images of size 42 × 14 and padding with zeros. For Channel 3, four 42 × 14 images are concatenated. Explicitly, the input to each of the three channels is as follows:

1. Channel 1 input = the Y channel of the resized (to 84 × 28) YUV converted image.
2. Channel 2 input = the Y, U and V channels of the 84 × 28 image resized to 42 × 14, concatenated and zero padded to achieve the overall dimensions of 84 × 28.
3. Channel 3 input = three edge maps (horizontal and vertical) obtained from each channel of the YUV converted image using a Sobel edge detector, resized to be 42 × 14 and concatenated along with the maximum values of these three edge maps into an image of overall size 84 × 28.

**Figure 4:**
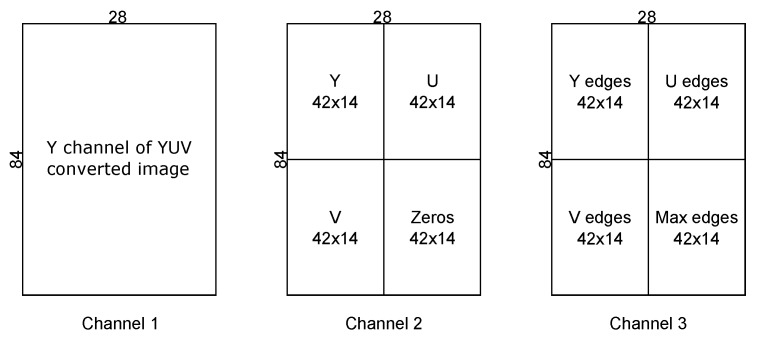
Composition of input channels to the pedestrian detector.

See Figure 4 for further clarification.

It should be noted that the pedestrian detector deep network uses a binary softmax function and therefore returns two values: the first value is the probability of the image window containing a pedestrian, and the second is the probability that it does not. We only use the former and simply ignore the latter.

As with most likelihood functions, the pedestrian detector-based likelihood function requires tuning in order to achieve the desired behavior. However, it only requires the adjustment of one parameter *γ*. Adjusting this parameter is straightforward and relatively simple. Higher values for *γ* result in higher likelihood values for all observations, and lower values result in lower likelihood values.

## 5. Experiments and Results

We evaluate the performance of the multi-Bernoulli filter with and without the interactive likelihood in a number of publicly available datasets and obtain quantitative results using standard, well-known metrics. In all of our experiments, the determination of the parameters *ζ*, *σ* and *γ* is empirical, but no exhaustive or rigorous search is employed; doing so could further improve our results. A summary of the experiments is provided here:

1. 2003 PETS INMOVE: (the 2003 PETS INMOVE dataset was originally obtained from ftp://ftp.cs.rdg.ac.uk/pub/VS-PETS/) In this dataset, the performance of the multi-Bernoulli filter without (MBF) the ILH, with the ILH (MBFILH), an implementation of the multiple hypothesis tracking (MHT) method [67], the multi-Bernoulli filter without the ILH and with a fixed target size (MBF FS), and the multi-Bernoulli filter with the ILH with a fixed target size (MBFILH FS) is evaluated; the HSV-based likelihood function in Equation (8) is used for all RFS filter configurations (MBF, MBFILH, MBF FS and MBFILH FS) within this dataset.
  - Empirically-determined interactive likelihood parameters: *ζ* = 0.15 and *σ* = 5.
2. Australian Rules Football League (AFL) [68]: In this dataset, the MBF and the MBFILH filter configurations use the likelihood function in Equation (8).
  - Empirically-determined interactive likelihood parameters: *ζ* = 0.15 and *σ* = 5 in reduced resolution images and *ζ* = 0.15 and *σ* = 10 in full resolution images.
3. TUD-Stadtmitte [69]: in this dataset, the pedestrian detector-based likelihood function in Equation (10) is used with the multi-Bernoulli filter without the ILH (MBF PD) and with the ILH (MBFILH PD).
  - Empirically-determined interactive likelihood parameters: *ζ* = 0.45 and *σ* = 150.
  - Empirically-determined pedestrian detector parameters: *ζ* = 0.30.

It is an intentional choice to not use the most recent benchmarks [55,56] because the framework (using standard detections) is not well suited for techniques that use SMC implementations and precludes track-before-detect methods in general.

### 5.1. 2003 PETS INMOVE

We first present results in the 2003 PETS INMOVE dataset. This dataset consists of 2500 frames of a soccer match. We use reduced resolution images (320 × 240) from the dataset to perform the evaluation. This illustrates the flexibility of our approach, as it is able to perform well in low signal-to-noise ratio (SNR) situations. Using reduced resolution also slightly reduces computation time.

Our implementation of the multi-Bernoulli filter is based on the source code kindly provided by the authors of [27]. Except for a few minor changes in the birth model parameters and color histogram computation, both implementations are identical. All trackers in this experiment are set up to track only the players on the red team (Liverpool) in the 2003 PETS INMOVE dataset. Within the RFS filters, targets are modeled as rectangular blobs and have states corresponding to Equation (2) with the *u* and *v* position of the target along with the width *w* and height *h* of the target’s bounding box. Because the implementation of the MHT in [67] does not estimate target size, we also evaluate the multi-Bernoulli filters with fixed target size.

In order to show that the proposed interactive likelihood is able to almost entirely eliminate the need for data association, we compare our method to the multi-Bernoulli filter proposed by Hoseinnezhad et al. in [27] without the data association algorithm [47] in place and to the MHT implementation of Antunes et al. [67]. Given the stochastic nature of the algorithms, a Monte Carlo evaluation was necessary. Specifically, we carried out 20 experimental trials for each filter configuration (MBF, MBFILH, MHT, MBF FS and MBFILH FS). We initialized the multi-Bernoulli filters with the targets in the first frame in order to achieve a fair comparison to the window scanning detector in the implementation of the MHT filter. Each trial progressed through all 2500 frames of the dataset.

**Figure 5:**
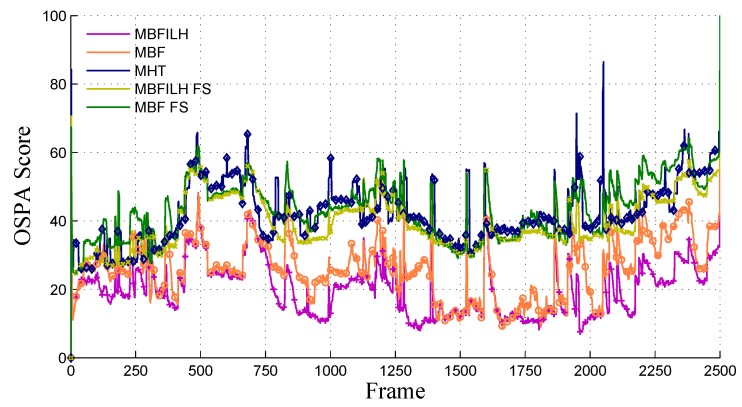
Average optimal sub-pattern assignment (OSPA) scores for all filter configurations over 2500 frames in the 2003 PETS INMOVE dataset. A lower score corresponds to better performance, as the OSPA metric measures the distance between estimated target tracks and the ground truth.

The optimal sub-pattern assignment (OSPA) [70] and CLEAR MOT (classification of events, activities and relationships for multi-object trackers) [71] metrics were used to obtain quantitative results from each filter configuration. In the OSPA evaluation, a cutoff parameter of *c* = 100 was used along with an order parameter of *p* = 1. See [70] for detailed information on the OSPA metric along with an interpretation of the parameters *c* and *p*. Briefly, *c* corresponds to the maximum allowed distance for two tracks to be considered comparable, and *p* determines how harshly outliers (tracks that are farther away than the cutoff) are penalized. As *p* increases (with *c* fixed), the metric penalizes outliers more severely. Figure 5 shows the average OSPA over the 20 trials at each of the 2500 frames. This graph shows that the underlying behavior of the multi-Bernoulli filter is not altered with the addition of the ILH term, and the OSPA score is, on average, lower with the ILH than it is without it.

**Figure 6:**
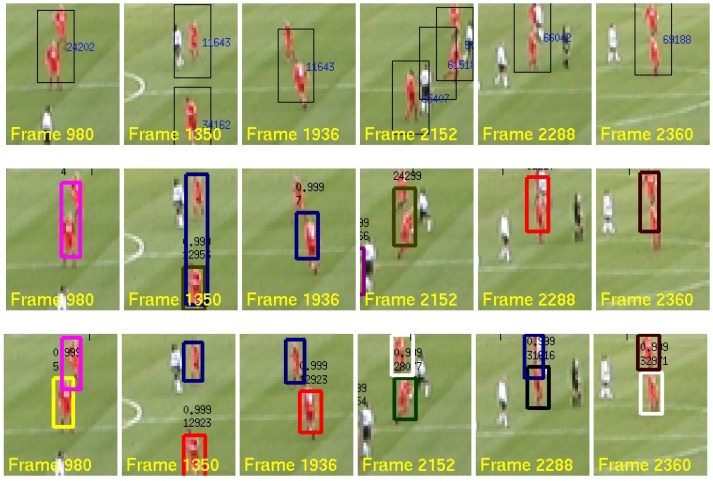
Illustrative scenarios in which the OSPA scores of the trackers under consideration differ significantly as is visible in Figure 5. The top row shows MHT results; the middle row shows MBF results; and the bottom row shows MBFILH results all in the low resolution 2003 PETS INMOVE dataset. The numbers in green in the top row correspond to the object identifiers for the MHT. The numbers in black above the targets in the second and third rows correspond to the target identifier, as well as the MBF estimated confidence level. In Frames 980, 2288 and 2360, both MHT and MBF incorrectly associate two targets with a single estimate. In Frame 1350, the MBF allows the estimate for one target to include a separate target, which was already correctly being tracked. In Frame 1936, MHT again merges two targets, and MBF finds only one. Frame 2152 shows that MHT incorrectly estimating one target in the region among three players. In all of these scenarios, the MBFILH correctly estimates all of the targets.

The mean OSPA scores of the average Monte Carlo trials in Figure 5 are summarized in Table 1. The results among the filter configurations that do not estimate target size (MBF FS, MBFILH FS and MHT) are all comparable. The MBF FS performs about 3% poorer than the MHT, while the MBFILH FS performs about 5% better than the MHT. It is expected that the configurations that estimate target size (MBF and MBFILH) achieve significantly higher performance than the filters that do not (MBF FS, MBFILH FS and MHT). It is somewhat surprising, however, that the MBFILH achieves a reduction of approximately 22% on the average OSPA score in comparison with the MBF. Because the OSPA metric does not incorporate labeling/track association errors, these results suggest that the ILH may also improve the overall tracking accuracy of the multi-Bernoulli filter. This is most likely due to fewer merging errors occurring with the ILH than without it; therefore, cardinality errors are significantly less frequent. As can be seen in Figure 5, there are certain frames where there are noticeable differences in OSPA scores for the different filter configurations. In order to illustrate why these differences exist, we show a selected number of these frames in Figure 6.

We evaluate the same 20 previously discussed Monte Carlo trials from the PETS dataset using the CLEAR MOT metric (CLEAR MOT source code was obtained from Andrew D. Bagdanov, Alberto Del Bimbo, Fabrizio Dini, Giuseppe Lisanti and Iacopo Mas at https://github.com/glisanti/CLEAR-MOT). For details on the CLEAR MOT metric, see [71]. We used a distance threshold (ratio of intersection to union of the area of the target’s bounding box to the area of the ground truth bounding box) of 0.1. This threshold is restricted to values between zero and one and is used to determine when a correspondence can no longer be made between the estimate and the ground truth, and the error is then labeled a missed detection. The CLEAR MOT metric is comprised of the following components (↑↓ indicate when better performance is represented by higher and lower values, respectively):

1. FNR: false negative rate (↓).
2. TPR: true positive rate (↑).
3. FPR: false positive rate (↓).
4. TP: number of true positives (↑).
5. FN: number of false negatives (↓).
6. FP: number of false positives (↓).
7. IDSW: number of i.d. switches (↓).
8. MOTP: multi-object tracking precision (↑).
9. MOTA: multi-object tracking accuracy (↑).

**Figure 7:**
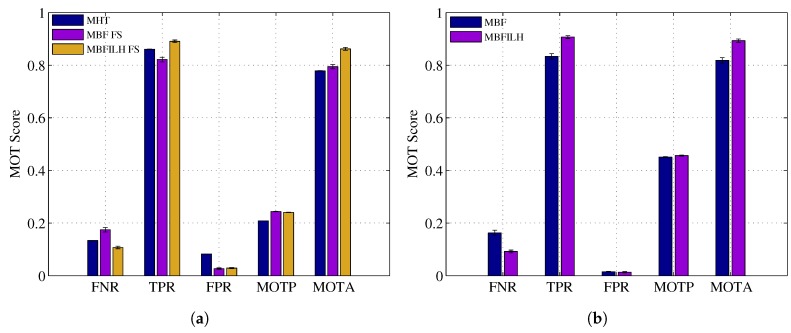
CLEAR MOT metric scores in the PETS dataset for (**a**) the MBF FS and MBFILH FS in the PETS dataset and (**b**) the MBF and MBFILH.

Results for the the 20 Monte Carlo trials for each filter configuration are summarized in Table 2. The FNR, TPR, FPR, MOTP and MOTA scores are shown in Figure 7a,b along with the corresponding standard deviations to illustrate the variability in the trials.

The largest improvement observed from the addition of the ILH was in the IDSW and MOTA metrics. Over the 20 trials and 2500 image frames, the MBFILH FS achieves 32 (approximately 49%) fewer identity switches than the MBF FS and 71 (approximately 68%) fewer than the MHT. In addition, the MBFILH FS yields an MOTA score of 86.2%, while the MBF FS and MHT MOTA scores are 79.4% and 77.8%, respectively. Superior performance is again seen in the MBF and MBFILH configurations. The effect of the ILH is even more pronounced in comparing the MBF and MBFILH. The MBFILH reduces the number of identity switches seen in the MBF by approximately 68% and increases the MOTA score from 81.8% to 89.3%. Because these two metric scores are directly influenced by labeling/i.d. error, the observed increases in these performance metrics suggest that the ILH term is able to significantly reduce the need for data association.

### 5.2. Australian Rules Football League

The second dataset we considered was the AFL dataset presented by Milan et al. in [68]. This dataset consists of 299 frames of an Australian Rules Football league match. Milan et al. explain that the AFL dataset is especially challenging for two reasons: (1) there is regular and frequent crowding of targets; and (2) contact and overlap among targets is common. We evaluate the ILH in this dataset using both reduced resolution images (320 × 240) and, for direct comparison to the results presented in [68], full resolution images (842 × 638).

We trained the MBF and MBFILH configurations to track all players and performed 15 Monte Carlo trials with the 320 × 240 resolution. We then performed another 20 Monte Carlo trials with the 842 × 638 resolution evaluating only the MBFILH.

**Figure 8:**
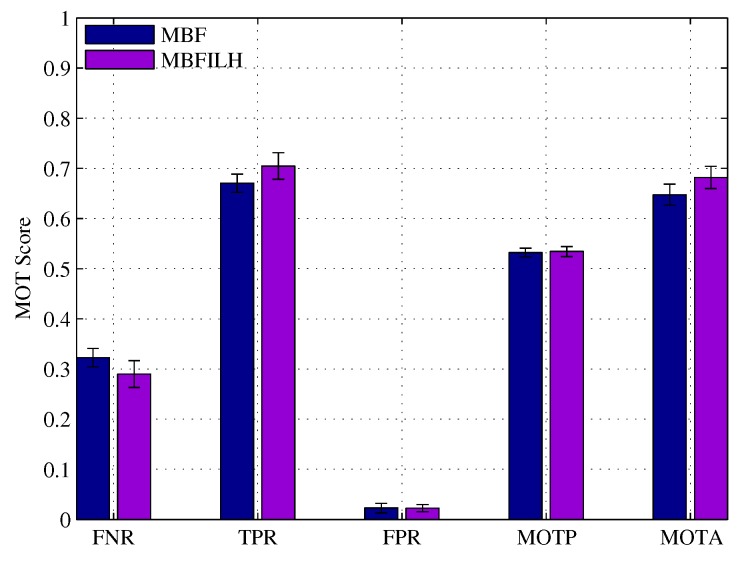
CLEAR MOT metric scores for the MBF and MBFILH in the reduced resolution Australian Rules Football League (AFL) dataset.

The CLEAR MOT metrics were used to evaluate the performance of each configuration in the reduced and full resolution trials. Again, a distance threshold of 0.1 was used. FNR, TPR, FPR, MOTP and MOTA scores, along with standard deviations, are shown for the reduced resolution trials in Figure 8. Full resolution results are compared in Table 3 to the results presented in [68].

As expected, the results are generally lower (for both reduced and full resolution trials) in this more challenging dataset than they were in the 2003 PETS INMOVE dataset. Despite overall lower scores, in the reduced resolution trials, the ILH term still reduced the number of identity switches of the MBF, on average, by four (approximately 22%) and increased the MOTA score from 64.8% to 68.2%.

**Figure 9:**
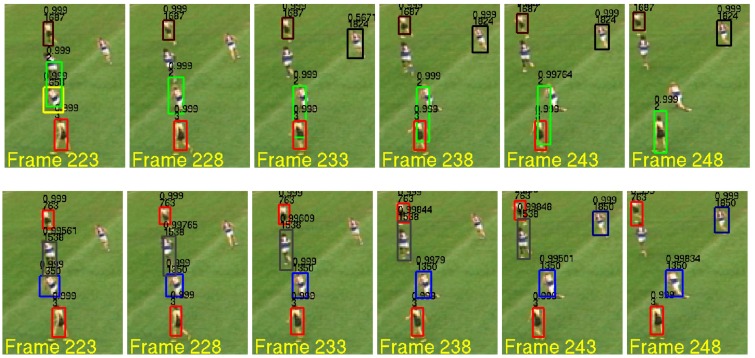
This is a particularly challenging sequence of low resolution AFL image frames (223 to 248). There are numerous overlapping and interacting targets. The top row are MBF results, and the bottom row are MBFILH results. Note that in Frame 223, the MBF drops a target (the one with the yellow bounding box), while the MBFILH does not. Furthermore, in Frames 233 to 243, the MBF target with the green bounding box starts to drift towards the target with the red bounding box, before finally merging in Frame 248, while the MBFILH is able to track these targets without drifting or falsely merging targets. However, the MBFILH does take longer to track the target farthest to the right in the images.

Some qualitative results from the reduced resolution trials are shown in Figure 9. The sequence of images consists of five targets interacting in close proximity, presenting a difficult tracking scenario for any multi-target tracker. The performances of the MBF and MBFILH are qualitatively compared. While both configurations fail to correctly track all five targets, the MBFILH is better able to track more of the targets in extremely close proximity. See the caption of Figure 9 for a more detailed explanation.

The multi-Bernoulli filter, with and without the ILH, consistently estimated the size of the targets within the AFL dataset to be significantly smaller than the size within the ground truth annotations, probably due to the high concentration of color in the torso of the players (because the likelihood function is based on HSV histograms and the players’ torsos have the highest contrast with the background, the MBF is essentially only tracking the upper half of the players). In an attempt to remedy this effect, the size estimates of the MBFILH in full resolution images were adjusted with a constant offset; however, this did not entirely eliminate the problem and explains the relatively poor performance of the MBFILH with respect to the MOTP metric in the full resolution trials (52.8% compared to the best result of 64.1%).

Despite the unimpressive MOTP scores, the MBFILH scores extremely well in the full resolution dataset with respect to the MOTA metric. As illustrated in Table 3, the MBFILH achieves an MOTA score of 66.3%, and the next highest performing method achieves 41.4%.

### 5.3. TUD-Stadtmitte

We evaluate the performance of the ILH in a much different situation than the previously examined ‘sport player tracking’ type of scenarios. For this, we use the TUD-Stadtmitte [69] dataset, which consists of 179 images of real data as pedestrians navigate through a street. This dataset is challenging because there are severe and frequent occlusions, and the position of the camera allows for a wide range of target sizes (some targets are farther away and appear much smaller than targets that are closer), which illustrates another advantage of our approach: the ability to adapt to different target sizes online. We use full resolution images (640 × 480) for this evaluation. In order to track pedestrians in this dataset, we use the much more general pedestrian detector-based likelihood function in Equation (10) with the multi-Bernoulli filter without the ILH (MBF PD) and with the ILH (MBFILH PD).

We carried out 10 Monte Carlo trials for each filter configuration (MBF PD and MBFILH PD) and used the evaluation code made publicly available by Milan et al. in [55] to compute the evaluation metrics (source code for multi-object tracking evaluation obtained at https://motchallenge.net/devkit/). The different measures calculated in this evaluation are:

1. Rcll: recall, the percentage of detected targets (↑).
2. Prcn: precision, the percentage of correctly detected targets (↑).
3. FAR: number of false alarms per frame (↓).
4. GT: number of ground truth trajectories.
5. MT: number of mostly tracked trajectories (↑).
6. PT: number of partially-tracked trajectories.
7. ML: number of mostly lost trajectories (↓).
8. FP: number of false positives (↓).
9. FN: number of false negatives (↓).
10. IDs: number of i.d. switches (↓).
11. FM: number of fragmentations (↓).
12. MOTA: multi-object tracking accuracy in [0, 100] (↑).
13. MOTP: multi-object tracking precision in [0, 100] (↑).
14. MOTAL: multi-object tracking accuracy in [0, 100] with log10 (IDs) (↑).

**Figure 10:**
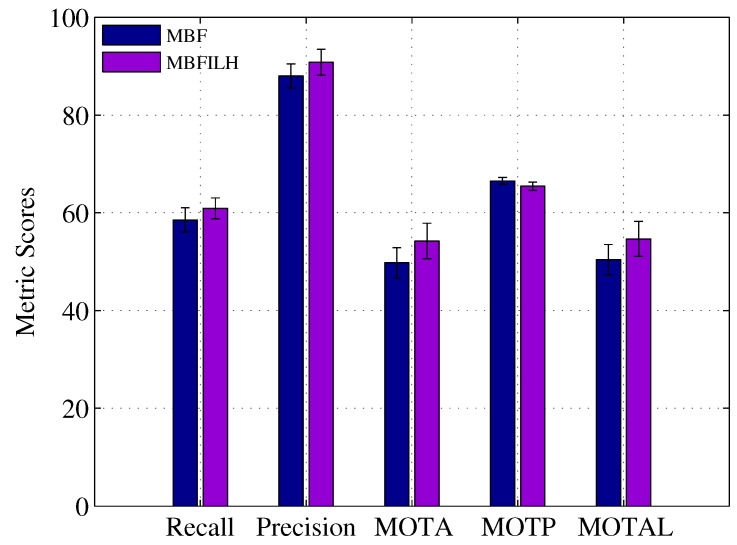
CLEAR MOT metric scores for the MBFILH PD in the TUD-Stadtmitte dataset.

Full results are presented in Table 4, and a selected number of CLEAR MOT metrics are shown in Figure 10.

**Figure 11:**
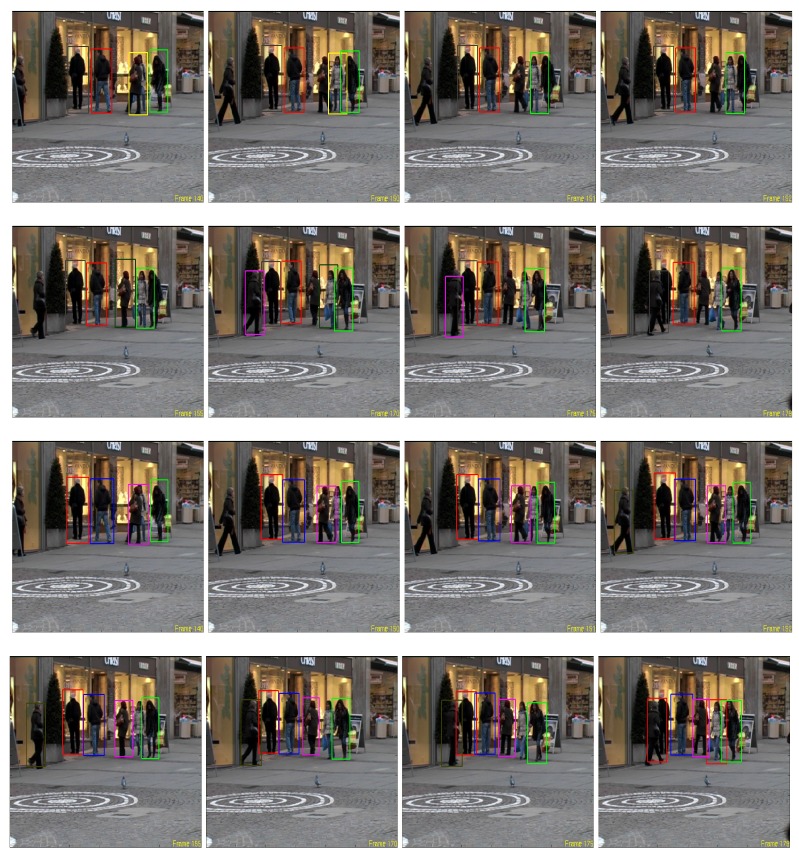
Tracking results for both the MBF PD (top two rows) and the MBFILH PD (bottom two rows) in the TUD-Stadtmitte dataset.

The ILH term improved the MOTA score of the MBF PD from 49.76% to 54.23%. In addition, the average number of i.d. switches dropped from 8.8 to 5.7 (approximately a 35% reduction). In fact, all scores were improved except for the ML metrics, which remained the same for both MBF PD and MBFILH PD, and the MOTP, which was slightly lower for the MBFILH PD (65.44%), than the MBF PD (66.53%). The reason for this slight decrease in MOTP performance is probably due to the ILH term forcing targets apart when they are extremely close. While this prevents unnecessary merging and i.d. switching, it may also cause estimates to be slightly shifted from the actual target. However, this is a relatively small decrease in MOTP performance, especially in comparison to the increases achieved in the MOTA, IDs and other metrics. Figure 11 shows some snapshots of the results on the TUD-Stadtmitte dataset.

## 6. Conclusions

In this paper, an interactive likelihood for the multi-Bernoulli filter was introduced. The interactive likelihood is a simple, yet effective way for reducing the need for data association. This is done by making the particle likelihood proportional to its distance (in pixels) to all other particles for all other existing targets. This allows for greater particle and target interaction. The most important feature of the proposed interactive likelihood is that it is constructed entirely within the RFS-Bayesian framework and therefore eliminates the need for heuristic ad hoc data association approaches.

The multi-Bernoulli filter augmented with the ILH term was also combined with a deep neural network pedestrian detector. Several experiments were performed using publicly available datasets (2003 PETS INMOVE, AFL and TUD-Stadtmitte) and well known metrics (OSPA and CLEAR MOT) in order to evaluate the performance of the various different filter configurations. Results indicate that the interactive likelihood term reduces the need for data association and increases the overall tracking performance of the multi-Bernoulli filter. Specifically, in all three datasets, the state-of-the-art RFS-based multi-Bernoulli filter saw accuracy improvements with the addition of the ILH term. The addition of the pedestrian detector makes the approach much more general, allowing the multi-Bernoulli filter to perform well in “real tracking” situations, such as that depicted in the TUD-Stadtmitte dataset.

Despite the observed improvements, there are some limitations to this approach, the most relevant of which is computation time. Even though, for a bounded number of particles, the computation time is *O*(*n*^2^), where *n* is the number of targets, in practice, a significant amount of time is spent calculating all of the distances between all particles. Therefore, plans for the immediate future are to investigate ways for increasing the overall speed of the algorithm. For example, due to their nature, the calculations are highly parallelizable and lend themselves to GPU implementations. Another potential way to achieve speed increases is to use more efficient algorithms for particle computation, such as quadtrees [74]. It should be noted, however, that distance computation is not the main bottleneck of the tracking algorithm as a whole. Sampling the image patches and computing their likelihoods is what dominates the computation time.

As mentioned in Section 4, the parameters of the interactive likelihood *σ* and *ζ* and of the pedestrian detector *γ* were all determined empirically. More sophisticated and rigorous search methods could be employed or approaches to automatically learn these parameters based on characteristics of the datasets could be developed. In particular, prior distributions parameterized by characteristics of the dataset (such as the video resolution) could be employed to estimate these parameters within a Bayesian framework. This would almost certainly result in improved performance.

Another limitation is that the RFS-based tracking methods require birth and death models. These models are often application specific and therefore must be changed or modified based on the application, which is cumbersome for obvious reasons. Hence, in order to make this approach much more general, situation independent birth and death models are necessary. This would also allow for a much closer comparison to the most current benchmarks [55,56]. Measurement-driven birth models, as proposed initially in [75] and later extended to the multi-Bernoulli filter for radar tracking applications in [76], provide a promising framework to address this problem.

Finally, our proposed approach does not take into consideration the extended target scenario in which a single target may generate multiple distinct measurements. Hence, scenarios involving, for example, extended concave targets [77] might still cause confusion. Although ad hoc solutions that take into consideration the expected size and concavity of the targets could be employed to overcome this limitation, a more principled solution would be to utilize RFS-based extended target tracking approaches, such as proposed in [78], while taking into account the expected distribution of the extended targets in the computation of the ILH. 

## Figures and Tables

**Table 1:** Mean OSPA scores of the average Monte Carlo trials for all filter configurations in the 2003 PETS INMOVE dataset. Best score(s) emphasized in bold. MHT, multiple hypothesis tracking; MBF, multi-Bernoulli filter; ILH, interactive likelihood; FS, fixed target size.

Method	Mean OSPA Scores
MHT	42.30
MBF FS	43.57
MBFILH FS	40.02
MBF	26.29
MBFILH	**20.39**

**Table 2:** Summary of 2003 PETS INMOVE CLEAR MOT metric scores. Best score(s) emphasized in bold. IDSW, number of label/i.d. switches; MOTP, multi-object tracking precision; MOTA, multi-object tracking accuracy.

Method	FNR	TPR	FPR	TP	FN	FP	IDSW	MOTP	MOTA
MHT	13.3%	86.0%	8.2%	14,789	2293	1415	104	20.9%	77.8%
MBF FS	17.4%	82.1%	2.7%	14,117	3004	465	65	24.4%	79.4%
MBFILH FS	10.7%	89.1%	2.9%	15,308	1846	496	33	24.0%	86.2%
MBF	16.3%	83.3%	1.5%	14,322	2803	264	62	45.1%	81.8%
MBFILH	**9.2%**	**90.7%**	**1.4%**	**15,583**	**1585**	**234**	**20**	**45.7%**	**89.3%**

**Table 3:** Summary of the full resolution AFL CLEAR MOT metric scores. Best score(s) emphasized in bold.

Method	MOTP	MOTA
SMOT [72]	60.8%	16.7%
DCO [73]	63.3%	29.7%
[68] (no init)	**64.1%**	32.0%
[68] (no LDA)	63.6%	39.0%
[68] (full)	63.6%	41.4%
MBFILH	52.8%	**66.3%**

**Table 4:** Mean metric scores for 10 trials of the MBF pedestrian detector (PD) and MBFILH PD in the TUD-Stadtmitte dataset. Best score(s) emphasized in bold where applicable. Rcll, recall; Prcn, precision; FAR, number of false alarms per frame; MT, number of mostly tracked trajectories; PT, number of partially-tracked trajectories; ML, number of mostly lost trajectories; FM, number of fragmentations; MOTAL, multi-object tracking accuracy in [0, 100] with log10.

Method	Rcll	Prcn	FAR	MT	PT	ML	FP	FN	IDs	FM	MOTA	MOTP	MOTAL
MBF PD	58.54%	88.00%	0.52	3.10	6.70	0.20	92.7	479.30	8.8	**12.10**	49.76%	**66.53%**	50.43%
MBFILH PD	**60.91%**	**90.79%**	**0.40**	**3.70**	6.10	0.20	**71.50**	**451.90**	**5.70**	12.90	**54.23%**	65.44%	**54.65%**
